# Single neuron electroporation in manipulating and measuring the central nervous system

**DOI:** 10.1186/1755-7682-3-28

**Published:** 2010-11-05

**Authors:** Ti-Fei Yuan, Manuel Menéndez-González, Oscar Arias-Carrión

**Affiliations:** 1 Neuroscience, NCI, Shanghai, 200001 China; 2 Unit of Neurology, Hospital Alvarez-Buylla, Mieres, Spain; 3 Experimental Neurology, Philipps University, D-35033 Marburg, Germany

## Abstract

The development and application of single neuron electroporation largely advanced the use of traditional genetics in investigations of the central nervous system. This quick and accurate manipulation of the brain at individual neuron level allowed the gain and loss of functional analyses of different genes and/or proteins. This manuscript reviewed the development of the technique and discussed some technical aspects in practical manipulations. Then the manuscript summarized the potential applications with this technique. Last but not least, the technique showed prospective future when combined with other modern methods in neuroscience research.

## Introduction

**Figure 1:**
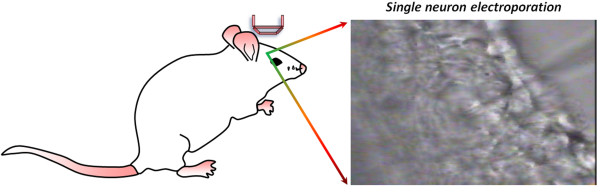
**Scheme of RNA interference (RNAi)-induced gene silencing by local electroporation in targeting in specific neuron**.

Many strategies have been developed to introduce charged or non-charged compounds into cell cytoplasm, including chemically modified dyes or peptides to cross the cell membrane (e.g. penetrating peptides), viral vectors or lipocomplexes for gene introduction [1,2]. A main defect of these techniques is the lacking of targeting accuracy that precludes specificity to given groups of cells. Electroporation is a suited method that allows the cellular introduction of small as well as large molecules [3,4]. The principle of electroporation is based on the assumption that the cell membrane, which was non-permeable to charged molecules in normal conditions, could form nano-sized pores under transmembrane electrical fields, thus permitting charged macromolecules to cross through the cell membrane (Figure 1) [5]. The transient pores then disappeared and the cells would return to a normal (i.e. not destabilized) condition within minutes as the electrical pulses ceased [6]. Following the first success on plasmid introduction to mammalian cells in 1982 [7,8], similar successes have been achieved on plant cells, bacteria cells, and yeasts [9].

Electroporation has, since the 1990 s, become a common laboratory technique for non viral transfection and molecule submission, however focused on bacteria or batch of cells in culture until the invention of in vivo electroporation - "modern electroporation". The most outstanding feature of this "modern electroporation" is the adoption of repeated charging with low voltage rectangular pulses, through which this approach could avoid the destroying currents to embryos or painful experiences to animals/human beings when high-voltage pulses were used. Developed on chick embryo [10,11], in vivo electroporation has now been successfully applied on liver, muscle, tumor, and skin for genetic material deliveries [12]. With stereotaxic apparatus facilitated microinjection, in vivo electroporation could target a defined small area in the adult brain [13]. This permits quick loading of genetic materials into specific areas of the brain without the needs to produce conditional knockdown/out animals. One intriguing application of in vivo electroporation in neuroscience is to perform molecule delivery on a single neuron scale for dissecting gain and loss of functions of specific genes and/or proteins. The present review first summarized the history of single neuron electroporation and discussed its mechanisms, followed with a discussion on its technical details, including the use of dyes and selection of proper voltage. Finally the review shed light on the advantages and potential application of this technique in modern neuroscience laboratories.

## The history of single neuron electroporation

Electroporation could be easily performed on isolated neurons *in vitro*, the process of which is similar to transfection of other cultured cells. People have succeeded in electroporation on isolated neurons or cultured neurons in vitro [14-16]. These studies tended to use microdevices with plate electrodes that create local and regular electrical fields for neurons to access the extracellular DNA plasmids or dyes, and are therefore applicable to a batch of neurons at the same time without the precise control at single cell scale. It is worth noticing that microfluidic devices that load a single neuron at a time with fixed electroporation parameters could be ideal for in vitro single neuron electroporation in the future [17,18].

In 2001, some neurophysiologists adopted micropipettes that have been used for electrophysiological recording for centuries to apply electrical pulses that electroporate plasmids into single neurons from the brain of intact Xenopus tadpoles or rat hippocampal slices in vivo [19]. Since the micropipette used for electroporation is filled with the substance or substances to be electroporated, the control of electroporation at single neurons scale is achieved by limiting both the dyes/DNA and the electrical field to the tip of the micropipette [20,21]. The detailed procedures are now available online, such as Nature Protocols [22], and some others in video formats [23,24].

During the process of "in ovo electroporation" (which means the electroporation on chicken embryo), Indian ink was injected under the chick embryo to create the shadow-contrast in visualizing the neural tube of the embryo. Similar ideas also emerged recently in single neuron electroporation on adult brain tissues. Because the two-photon microscopy requires pre-labeling of neurons with fluorescence protein, either by transgenic performance or viral-transfection; Kitamura *et al*. (2008) developed the method of shadow-patching/electroporation by loading dyes (e.g. Calcium indicator dyes or fluorescent dyes) in to extracellular spaces of neurons, thus creating contrast between "dark" neurons and "bright" backgrounds, for single neuron targeting and electroporation [25]. While the dyes would be cleaned by circulation, the gene expression can be stable for months afterward and be tracked for long-term imaging [22]. This advance further expanded the application of single neuron electroporation to neurons with identified physiological functions, such as calcium dynamics, illuminating the links between network dynamics and connectivity.

## Setting up the single neuron electroporation system

The successful establishment of a single neuron electroporation system should consider variances caused by resistance of tissues or solutions in which neurons are suspended, the material nature of the electrodes, the molecular weight and charge of the substances to deliver, and most importantly, the endurance of the tissue to electrical currents. Furthermore, because of the different underlying principles of *in vitro *electroporation and *in vivo *electroporation, the settings are completely different. The following paragraphs are going to address some most basic principles in setting up the single neuron electroporation system.

The efficiency of *in vitro *neuron electroporation largely depends on cell density, the proper voltage and pulse duration and the presence of calcium ions in buffer used for electroporation [26-29]. The temperature and the general composition of electroporation buffer can also have minor effects on the transfection success [26]. Put in brief, a higher cell density is required for neuron (e.g. 10^9 ^forebrain neurons per ml), compared to other types of cells (e.g. 10^7 ^HeLa cells per ml) [30] (Though higher cell density can be used, it may cause increased incidences of cell fusion); high voltage in short pulse form should be adopted, above a thousand V/cm lasting for only several ms. It is worth mentioning that the repeated pulses as what was used for bacteria electroporation leads to substantial neuron death; The free calcium ion at 0.1-1.0 mM in electroporation can greatly enhance the re-sealing of cell membrane [31], therefore the addition of CaCl_2 _can increase the survival rate as well as the transfection efficiency simultaneously. At the same time, a very high temperature generated by the electrical currents can lead to cell death, and a prior electroporation low-temperature incubation can help [32]. Finally, the ionic strength and the resulted resistance of electroporation buffer solution should be considered.

Parameters for *in vivo *electroporation are largely different from what was described in last paragraph. For example, the chicken embryos were manipulated under 20-25 V with five 50 ms pulses [11], with a much lower level of voltage compared to suspended cell solution which greatly enhances the embryo viability, though a higher voltage (e.g. 60 V) can still be adopted to promote the efficiency of gene transfer in some cases [33,34]. For micro-pipette based single neuron electroporation, a stimulator, an oscilloscope, and a micropipette holder with micro-manipulator is required [35]. The voltage to be used can be as low to 5-10 V or determined through examination of current amplitude on the oscilloscope [22,23,25,35] depending on the development stages and the substances to be delivered. It is suggested that one start with fluorescent dyes for electroporation to allow a fast feedback of parameter settings in one's own system. Tadpole and Zebrafish larvae are small, transparent and easy to be manipulated under the microscope, providing two of the most useful animal models to practice with.

## Advantages of single neuron electroporation

Single neuron electroporation permits the transfection of multiple genes using plasmids at the same time, without the needs of production of several viral vectors [36]. As an example, the gene transfer of light-activated channels has been successfully used to excite specific neurons in barrel cortex [37]. It is also possible to deliver DNA plasmids encoding other types of ion channels, siRNA or proteins into single neuron for functional analyses, (a regenerating axotomized neuron, for example) [38-40]. It is conceivable to perform genetic function analysis, gene therapy, and molecule submission at a single neuron scale now (Figure 1).

One obvious advantage of this approach is the possibility to perform electrophysiological or functional analyses shortly before and/or after the electroporation [41], given that the electroporation process itself has little effect on electrophysiological properties of the neuron [42]. This is helpful in dissecting genetic functions in local circuits within brain slices, or to set up a proper system for genetic therapy for brain disorders.

Another intriguing point for this technique is to label single neuron in vivo by loading fluorescent dyes or fluorescent protein-encoded proteins into the soma. This permits the identification of single neuron in vivo; it is also possible to image microstructure-based plasticity, for instance, by loading the neuron with calcium indicator fluorescent dyes [43-45]. This approach thus greatly expanded the technological repertoire of cell functional analyses using high resolution imaging.

Thirdly, It is possible to increase the scale of electroporated area, simply by enlarging the tip diameter or changing the position of the micropipette, and perform repeated electroporation, leading to the sparse and random labeling of a subset of neurons near to the tips with a "Golgi-like staining" pattern [45]. On the other hand, micropipette based single neuron electroporation has also been proved to be effective on very small individual neurites (<2 μm) and single growth cones [46], which are not eligible for microinjection due to the size restriction.

Because of the fact that neurons are difficult to transfect, electroporation provides one of the best approaches in non-viral transfection methods. Current advances on the understandings in principles of electroporation and electrophysiological techniques have facilitated the emergence and development of single neuron electroporation technique. Besides all the advantages of electroporation compared to other gene transfer methods discussed above, single neuron electroporation is the best approach so far for substance delivery at single neuron or circuit level.

## Future applications of single neuron electroporation

Electroporation was traditionally recognized as a way of substance delivery into the cell. Therefore one important standard in judging the application of single neuron electroporation is the variety of substances that can be delivered. In the past, dyes, heavy metal ions, genetic materials, proteins (including enzymes and antibodies), and many chemicals have been tested for their potential to be used in electroporation [9]. The general in vivo electroporation to the brain with applications on gene screening (gain- and loss- of function) and gene therapy have already been put into practice [13]. The present review, however, will focus on the discussion of potential application on why and when the brain electroporation should be conducted at a single neuron level.

In the area of developmental neurobiology, single neuron electroporation can provide the unique chance for cell lineage analyses in brain development. Currently most available cell labeling methods such as the use of diffusion dyes lack the specificity of single cell labeling, and therefore are only used in a limited way for monitoring of batch cell migration. It is still hard to specify the interaction and communication of single neuron/neuroblast with other cells during its migration and specification following birth (Figure 1). The manipulation of cell surface protein, the expression of ion channels, neurotransmitter receptors or labeling of the cell with fluorescent dyes/proteins through single neuron electroporation can thus greatly improve our understanding of brain development and postnatal neurogenesis [47,48]. In the adult brain, the activity of single neuron transfected with fluorescent proteins or light-controlled ion channels can be artificially controlled during and after the maturation of these new cells [49]. Thus it is possible to analyze the single neuron function in behavior control [37], and to understand the coding at the oligo-population level with restricted electroporation to a small region in the neural stem cell niche [50].

In the area of neuroanatomy, the technique of loading fluorescent dyes into a single neuron without intracellular injection is exciting news for neuroanatomists who are interested in tracing single axon over long distances with high resolution imaging during development and regeneration [51] Currently such works have only been performed on cultured slices due to the transient gene expression following plasmid electroporation [52-54]; the use of genome-integrating viral vectors may expand the application to live animals for imaging over a relatively long period.

## Questions remain

The amazing progresses in single neuron electroporation, however, have also left some unsolved problems. First of all, any approach that requires visualization for in vivo single neuron manipulations are currently limited by the depth of microscopy. Hopefully, with the facilities of multi-photon microscopy, or microendoscopy [55], people can visualize much deeper neurons inside the whole brain for further manipulation. The shadow contrast visualization could also be improved with new types of fluorescent dyes. Furthermore, when combined with electrophysiological recording, so-called "blind" single-electrode electroporation in vivo would be possible following established techniques for "blind" extra- or intra-cellular in-vivo recordings with patch electrodes [41].

Secondly, parameters that maximize the submission of different molecules and cell viability simultaneously are currently limited, which could only be adopted from previous studies on optimal electroporation parameters in other systems. Thirdly, compared to intracellular injection, the loading of dyes through electroporation has the disadvantage of unknown intracellular dye concentration after dye loading [43], which brings out the need to use dual/combined indicators or other methods for quantitative computation. Nevertheless more and more practices with single neuron electroporation in CNS investigation would finally improve this technique and widen its applications in coming future.

## Competing interests

None of the authors have actual or potential conflict of interest including any financial, personal or other relationships with other people or organizations that could inappropriately influence, or be perceived to influence, our work.

## Authors' contributions

TFY and OAC designed, conducted the literature review and drafted most of the manuscript. MMG performed the literature review and the drafting of the manuscript. All authors were equally involved in reading and approving the final manuscript.
